# Methodology for Evaluation of Residual Stress Effect on Small Corner-Crack Initiation and Growth

**DOI:** 10.3390/ma12182904

**Published:** 2019-09-09

**Authors:** Janghwan Kim, Jun Won Kang, Dong-Eun Lee, Dae Young Kim

**Affiliations:** 1 Department of Civil Engineering, Gwangju University, Gwangju 61743, Korea; 2 Department of Civil Engineering, Hongik University, Seoul 04066, Korea; 3 School of Architecture and Civil Engineering, Kyungpook National University, DaeGu 41566, Korea; 4 Department of Architectural Engineering, Pusan National University, Busan 46241, Korea

**Keywords:** corner crack, residual stress, fatigue crack growth, aluminum 6061-T6

## Abstract

The growth behavior of a naturally initiated corner crack under a uniform residual stress field is investigated in this study. A convenient method is proposed to induce and evaluate the uniform residual stress field for a beam-type specimen. Fatigue tests are conducted with a rotary bending fatigue machine to investigate the growth of the corner crack. For this reason, a cylindrical specimen, which is typically used for rotating bending tests, is modified into a beam specimen. The corner crack growth behavior under residual stress is evaluated based on linear elastic fracture mechanics (LEFM) and compared with long through crack data. The test results verify that the corner crack growth under residual stress can be effectively evaluated by LEFM and estimated using long crack data.

## 1. Introduction

Residual stress can be introduced into a structure by various manufacturing processes such as forging, welding, and cold drawing. It can significantly impact the fatigue lifetime of a structure. Compressive residual stress increases fatigue lifetime by delaying crack growth, and tensile residual stress reduces fatigue lifetime by expediting crack initiation and propagation [1,2]. To accurately estimate the fatigue lifetime, therefore, the residual stress needs to be evaluated correctly. For this reason, significant amount of effort has been made by researchers to quantitatively evaluate the residual stress.

Until now, research on artificially controlled residual stress has focused mainly on through cracks, which have initial notches, using compact tension (CT) specimens. By applying and releasing a tensile overload on a CT specimen, a residual stress field is induced near the crack tip [3,4]. This stress can be calculated when a relevant residual strain distribution is known. However, it is difficult to find such a distribution near the crack tip in CT specimens. Schindler [5] proposed a relatively simpler method to estimate the residual stress based on the relationship between the fracture mechanics and a stress intensity factor. To utilize this method, however, the stress intensity factor should be determined in advance, experimentally. Therefore, a detailed finite element analysis, including material nonlinearities, is necessary to determine the residual stress profile.

In this study, a convenient method is introduced to induce a uniform residual stress on a specimen by the process of bending. The applied residual stress is evaluated based on the inelastic bending theory. Additionally, the initiation and growth of a corner crack is investigated using the uniform residual stress field. Cyclic loads are applied with a rotating bending fatigue test. For this test, a cylindrical specimen, which is typically used for the rotating test, is modified into a square-shaped specimen. To detect the initiation and growth of the corner crack, a plastic replication method is utilized. The growth behavior of the naturally initiated corner crack is then evaluated based on LEFM and subsequently compared with long through crack data.

## 2. Residual Stress on a Specimen

### 2.1. Specimen Modification

**Figure 1:**
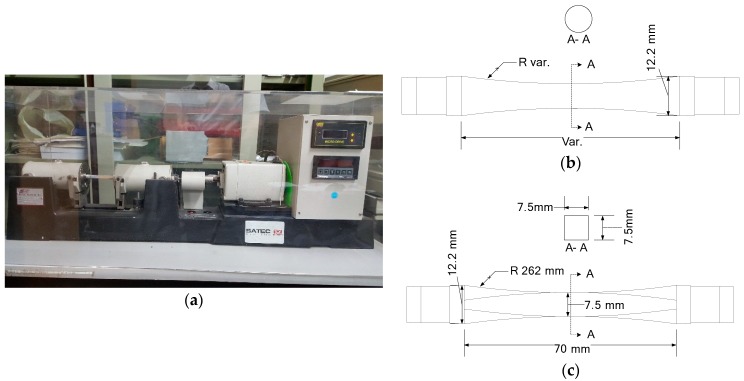
Rotary specimen. (**a**) R.R. Moore high-speed fatigue test machine; (**b**) cylindrical rotary specimen; (**c**) rotary specimen modified to have a square shape.

The initiation and growth of corner cracks under a uniform residual stress field are investigated using a modified rotary bending specimen made of Aluminum 6061-T6. Cyclic loadings are applied with the R.R. Moore high-speed fatigue test machine as shown in Figure 1a. A cylindrical specimen, which is typically used in rotating bending fatigue test (as shown in Figure 1b), is machined to form a smooth square beam in the central region (as shown in Figure 1c) to investigate the corner crack initiation and growth behavior and subsequently introduce a uniform residual stress field. The length and width of the square beam section in the modified specimen are 10 mm and 7.5 mm, respectively.

### 2.2. Residual Stress Application on a Beam

**Figure 2:**
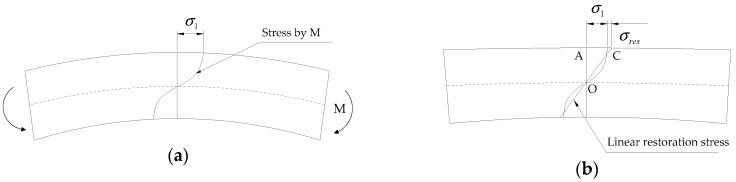
Procedure to induce residual stress on the top and bottom surface of a specimen. (**a**) Loading with overload moment *M*; (**b**) linear restoration by unloading *M*.

The residual stress can be introduced on the top and bottom surfaces of a beam by applying an overload moment (i.e., a moment inducing an inelastic stress on the surface of the beam) and subsequently releasing it, as shown in Figure 2. When the overload moment, *M*, as shown in Figure 2a, is applied to the beam, the stress exceeds the elastic limit, thereby resulting in a plastic strain on the beam surface. By releasing the applied moment, the bent beam starts to be linearly restored owing to the interior elastic portion of the material. The full restoration, however, is deterred as a result of the permanent deformation caused by the plastic strain. Consequently, a uniform residual stress along the width of the beam is induced at the top and bottom surfaces, as depicted in Figure 2b. 

Nadai [6] analyzed the inelastic bending problem of a rectangular beam that can be applied to a modified specimen, using the upper and lower surface strains. He proposed a closed form of the surface stress in terms of the applied overload bending moment *M* and corresponding surface strain *ε*_1_, as presented in Equation (1):

(1) $$\sigma_{1}=\frac{2}{wh^{2}}\left(2M+\varepsilon_{1}\frac{dM}{d\varepsilon_{1}}\right)$$

where *M* is the overload bending moment; *σ*_1_ is the surface stress of the rectangular beam induced by *M*; *ε*_1_ is the surface strain corresponding to *σ*_1_; *w* is the width of the beam; and *h* is the height of the beam. 

The beam is linearly restored by releasing *M* and a residual stress is generated, as shown in Figure 2b. The residual stress can be computed using a moment equilibrium. Half of *M* is equal to the sum of internal stress over the area OAC in Figure 2b, as shown in Equation (2). By rearranging the equation, the residual stress, *σ_res_*, can be obtained in terms of *M* and *σ*_1_, as shown in Equation (3).

(2) $$\int_{0}^{h/2}\frac{\sigma_{1}+\sigma_{res}}{h/2}y^{2}wdy=\frac{1}{2}M$$

(3) $$\sigma_{res}=\frac{6M}{wh^{2}}-\sigma_{1}$$

**Figure 3:**
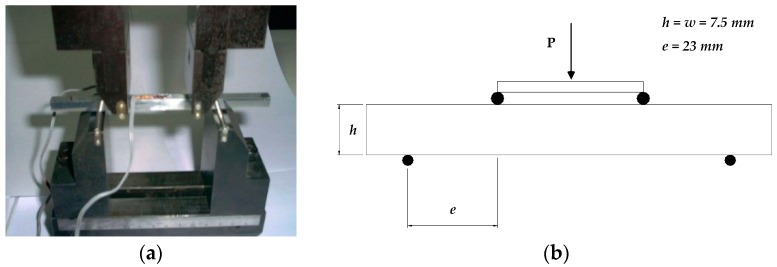
Four-point bending test. (**a**) Bending fixture; (**b**) schematic configuration of four-point bending test.

According to Equation (3), the residual stress, *σ_res_*, can be calculated once the overload bending moment *M* and corresponding surface stress *σ*_1_ are known. As indicated in Equation (1), *σ*_1_ is a function of *M* and *ε*_1_. Considering that *M* is dependent on *ε*_1_, *σ_res_* can be calculated for a specific overload bending moment when the relationship between *M* and *ε*_1_ is known. In this study, the applied bending moment *M* is assumed as a function of the surface strain *ε*_1_, and the relationship is obtained from data regression analysis based on four-point bending test results with total nine specimens, which were conducted according to ASTM E855 [7]. For the point-bending tests, square-shaped specimens were utilized with bending fixtures, as shown in Figure 3a. These fixtures were attached to a universal testing machine (UTM) to induce a pure bending moment on the specimen with an axial load *P*, as schematically described in Figure 3b. The axial load *P* was applied using the UTM by controlling the displacements. To measure the surface strain of the specimen, strain gauges were attached to the top and bottom of the specimen.

**Figure 4:**
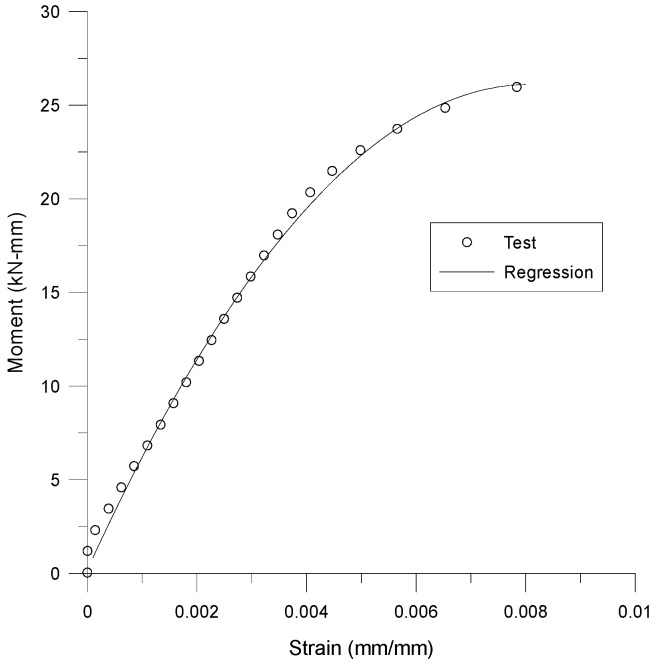
Test and regression results of moment-strain relationship.

Figure 4 shows the one of the test results in total nine four-point bending tests. The test results are fitted with a function from a data regression analysis. The second order polynomial in Equation (4) is used as the fitting function for the relationship between *M* and *ε*_1_. The coefficients of the polynomial are determined using the least square method. The regression results of each test are listed in Table 1; the average value of each coefficient is used to determine the moment function which is as follows.

(4) $$M(\varepsilon_{1})=c_{1}\varepsilon_{1}^{2}+c_{2}\varepsilon_{1}+c_{3}$$

### 2.3. Application and Evaluation of Residual Stress on Modified Specimen

To induce a uniform residual stress on the modified rotary specimen as shown in Figure 1c, the specimen is also bent with the four-point bending fixture used for the bending test conducted to find out the moment function. Note that the axial load, *P*, in Figure 3b should be higher than a load *P_y_* that induces the yield stress, *σ_y_*, on the surface of the specimen. The yield strength of Aluminum 6061-T6 is 240 MPa at room temperature for rectangular beams [8], and the corresponding axial load, *P_y_*, is 1415 kN, evaluated using the elastic bending theory in Equation (5). In this study, an axial load of *P* = 1825 kN (i.e., exceeding the elastic limit) is applied to induce residual stress on the surface of the modified rotary specimen. The bending moment, *M*, induced by the applied axial load, *P*, is 20.99 kN-mm with a 23-mm moment arm. The surface stress *σ*_1_ is 259.7 MPa, according to Equation (1), and the residual stress *σ_res_* is 38.8 MPa, calculated using Equation (3).

(5) $$\sigma_{y}=\frac{M_{y}}{S}=\frac{3P_{y}e}{wh^{2}}$$

*σ_y_* is the yield strength, *M_y_* the yield moment, *S* the elastic section modulus, *P_y_* the axial load corresponding to *M_y_*, *w* the width of the beam’s cross-section, and *h* the beam height.

## 3. Modeling of Corner Crack

### 3.1. Stress Intensity Factor Induced by Remote Forces

**Figure 5:**
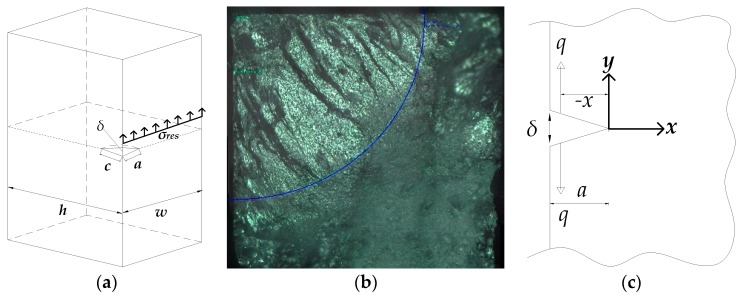
Corner crack on rectangular specimen. (**a**) Analytical crack model; (**b**) fractography of corner crack; (**c**) crack opening displacement by a point load *q*.

Newman and Raju [9] proposed a stress intensity factor equation (Equation (6)) which can be applied to elliptical cracks embedded in a rectangular plate, as shown in Figure 5a.

(6) $$K_{I}=\left(\sigma_{m}+H\sigma_{b}\right)F\sqrt{\frac{\pi a}{Q}}$$

where *σ_m_* is the stress induced by a remote tensile force and *σ_b_* the stress induced by a remote bending moment. *F* and *H* are boundary correction factors, *Q* is a shape factor accounting for the different shape of a crack depending on its aspect ratio; *a* is the crack length along the width direction, and *c* the crack length along the height direction.

In Equation (6), the shape factor *Q* depends on the crack aspect ratio *a/c*, which is shown in Figure 5a. *F* and *H* depend on the crack aspect ratio *a/c*, finite width *a/w*, finite depth *b/h*, and crack-front coordinates *Φ*. In this study, the rotary bending machine is used to apply a remote bending stress on the modified specimen. Therefore, only the bending moment is applied to the specimen and *σ_m_* = 0 in Equation (6). The value of *a/c* = 1 is assumed based on the fractography of the fractured specimen, as shown in Figure 5b. The width *w* and depth *h* of the specimen are both 7.5 mm.

### 3.2. Stress Intensity Factor Induced by Residual Stress

A stress intensity factor by the residual stress, *K_res_*, can be evaluated based on the crack opening displacement *δ*. As shown in Figure 5c, the crack opening displacement is induced by a point load *q* and can be calculated using Equation (7) [5]. Irwin [10] showed that the crack opening displacement could be expressed in terms of the stress intensity factor, which is shown in Equation (8). Assuming that *Gr* is a function describing the crack shape and boundary conditions, then the stress intensity factor for the quarter-elliptical-shaped crack in Figure 5a can be expressed by Equation (9). The details of *Gr* can be found in reference [11]. Note that the residual stress applied on the elliptical crack can be regarded as the sum of a point load *q* acting on an infinitesimal distance *dx*. Therefore, the stress intensity factor induced by residual stress can be calculated by integrating Equation (9) from 0 to the crack length *a*, as indicated in Equation (10).

(7) $$\delta =\frac{8q}{\pi E}\cosh^{-1}\left(\frac{a}{a-x}\right)H\left(\frac{a-x}{a}\right),\;H\left(\frac{a-x}{a}\right)=1.681-0.384{\left(\frac{a-x}{a}\right)}^{0.38}$$

(8) $$\delta =2\upsilon =\frac{4\sqrt{2}K_{I(q)}}{E}\sqrt{\frac{a}{\pi }}$$

(9) $$K_{I(q)}=\frac{\sqrt{2}P}{\sqrt{\pi a}}\cosh^{-1}\left(\frac{a}{a-x}\right)H\left(\frac{a-x}{a}\right)G_{r}$$

(10) $$K_{I(res)}=\frac{\sqrt{2}\sigma_{res}}{\sqrt{\pi a}}\int_{-a}^{0}\cosh^{-1}\left(\frac{a}{a-x}\right)H\left(\frac{a-x}{a}\right)G_{r}dx$$

## 4. Crack Growth Tests and Results

In this study, the effect of a residual stress on the initiation and propagation of a corner crack is investigated. For this purpose, crack growth tests are conducted for modified rotary specimens with and without uniform residual stress listed in Table 2. Cyclic fatigue loads are applied to the specimens using the R.R. Moore High-Speed Fatigue Test Machine. The surface stress on the specimens by the applied load corresponds to 47% of the ultimate strength, 310 MPa, with a loading frequency of 33 Hz. Cracks are detected using a plastic replication method that has high resolution of up to 0.1 µm [12]. The crack length is measured using a DZ2 Video Microscope with a 10× zoom and 50× magnification lens. To conduct plastic replication of the cracks, the fatigue tests are interrupted every 10,000 cycles until fracture of the specimens.

### 4.1. Growth Behavior of Corner Crack

The growth behavior of a through crack on a CT or CCT specimen has a sigmoidal form, comprising three distinct stages: stage I, II, and III. In stage I, short cracks are initiated by the formation of a slip band and coalesce into a major crack. As the crack continues to grow, it enters into stage II, where the crack growth rate da/dN shows a linear relationship with the stress intensity range *∆K* in a log scale plot. Stage III corresponds to a region where the growth rate of the crack increases rapidly and deviates from the linear trend. 

**Figure 6:**
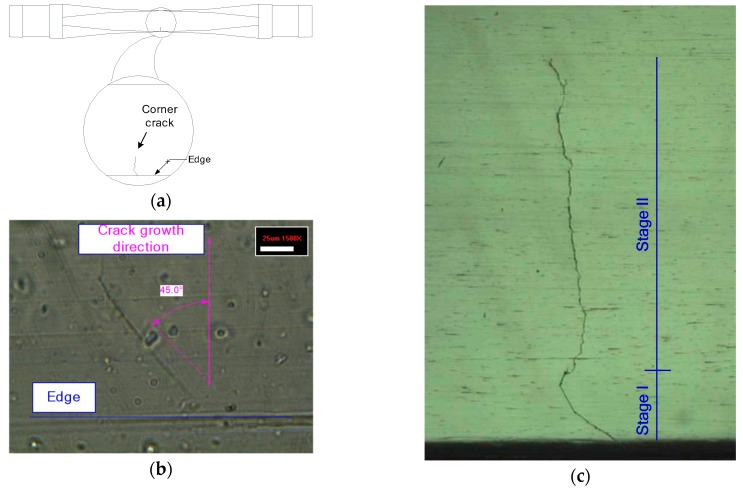
Corner crack initiation and propagation. (**a**) Crack location; (**b**) crack initiation; (**c**) crack propagation.

The initiation and propagation of a naturally occurring corner crack in the modified specimen follow the typical growth behavior of the through crack mentioned above. As shown in Figure 6a, the fatigue crack of the modified specimen is initiated near the edge where the stress concentration occurs and propagates into the plane. Figure 6b shows the crack initiation stage, where the crack grows very slowly along the plane inclined about 45 degrees to the normal stress direction by a bending moment. In this stage, it has been reported that the crack growth is dominantly affected by the crystallographic planes of the material [13,14]. Beyond this stage (i.e., stage I), the crack propagates along the plane perpendicular to the normal stress, as shown in Figure 6c photographed directly from the surface of the modified specimen. This stage corresponds to stage II, where the crack grows stably. When the crack length is approximately 40% of its width, it grows rapidly and ends with the complete fracture of the specimen. 

**Figure 7:**
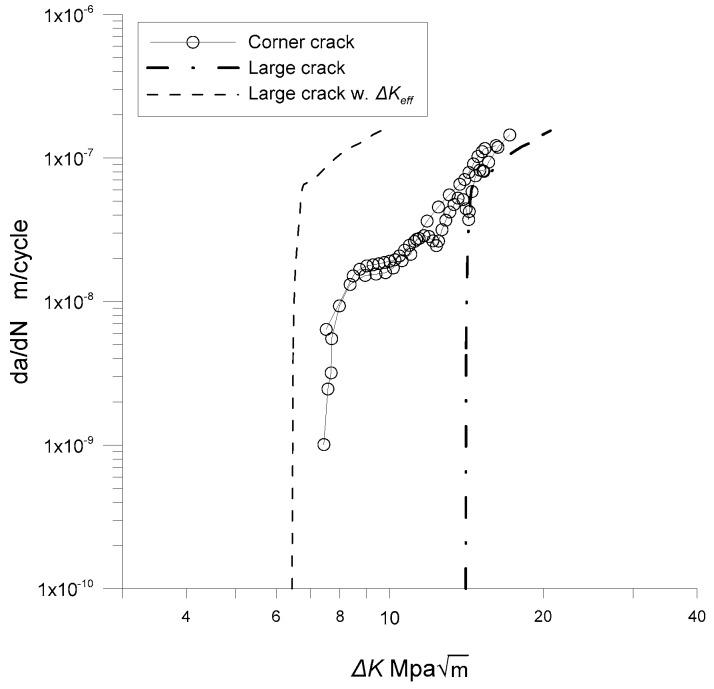
Growth rates of corner cracks.

Figure 7 shows the growth rates of corner cracks in the modified specimens induced by the rotary bending test. The growth behaviors of the corner cracks are compared with the long crack data of a CCT specimen collected from reference [15]. As shown in Figure 7, the naturally initiated cracks exhibit a finite growth rate even though the stress intensity range is below the *∆K_th_* of the long crack. Note that the measured initial crack lengths are approximately 100 µm, as shown in Figure 6b. Previous research indicates that at a low stress intensity factor near the threshold, *∆K_th_*, mechanically short cracks (i.e., with a crack size between 100 µm and 1 mm) not only grow much faster than long cracks but also have a finite growth rate below the threshold [16,17,18]. Considering that the corner cracks are in a range of mechanically short, the observed crack growth behavior near the threshold is consistent with previous research on mechanically short crack growth behaviors.

### 4.2. Crack Closure Effect on Long Crack Growth

As mentioned in the previous section, short cracks grow in range below the threshold. It has been reported that the long crack growth behavior is sensitive to a crack closure effect, especially near the threshold [19,20] and the plastic-induced crack closure (PICC) is the dominant phenomenon that affects the growth of long cracks [21]. For this reason, the crack closure effect is evaluated using a crack opening stress equation for the CCT specimen. Newman [17] proposed a term called opening stress, *σ_op_*, which is expressed as a function of a stress ratio *R* using Dugdale’s model, as shown in Equations (11) and (12).

(11) $$\frac{\sigma_{op}}{\sigma_{\max}}=A_{0}+A_{1}+A_{2}R^{2}+A_{3}R^{3}\;\mathrm{for}\;R\geq 0\;\mathrm{and}$$

(12) $$\frac{\sigma_{op}}{\sigma_{\max}}=A_{0}+A_{1}R\;\mathrm{for}\;-1\leq R<0,$$

where *σ_o_* is the average value between the ultimate strength and the yield strength of material; R = *σ_min_*/*σ_max_*, *σ_max_* is the maximum stress, and *σ_min_* is the minimum stress. The coefficients of the crack closure function are as follows:

(13) $$A_{0}=\left(0.825-0.34\alpha +0.05\alpha^{2}\right){\left[\cos\frac{\pi \sigma_{\max}}{2\sigma_{o}}\right]}^{1/\alpha }$$

(14) $$A_{1}=\left(0.415-0.071\alpha \right)\frac{\sigma_{\max}}{\sigma_{o}}$$

(15) $$A_{2}=1-A_{0}-A_{1}-A_{3},\;\mathrm{and}$$

(16) $$A_{3}=2A_{0}+A_{1}-1.$$

In Equation (13), *α* is a constraint factor for a crack in a specimen. Newman et al. [22] proposed an expression for the constraint factor in terms of the maximum stress intensity factor scaled by the thickness of a specimen, as shown in Equation (17); they also suggested values of *β* = 1.25 and *γ* = 0.85 for a CCT specimen.

(17) $$\alpha =1.15+\beta e^{-\gamma K_{n}^{1.5}},\;K_{n}=K_{\max}/\left(\sigma_{o}\sqrt{B}\right)$$

The crack closure effect for the CCT specimen is subtracted by evaluating the crack growth rate under the effective stress intensity by subtracting the opening stress from the maximum stress, as indicated in Equation (18). The dashed line in Figure 7 shows the crack growth rate without the crack closure effect. As seen in Figure 7, the naturally initiated corner crack shows a lower growth rate than the long crack once the crack closure effect is removed. In addition, the threshold of the corner crack is lower than that of the long crack.

(18) $$\Delta K_{eff}=\left(\sigma_{\max}-\sigma_{op}\right)\sqrt{\pi a}F_{cct}\;\mathrm{and}$$

(19) $$F_{cct}={\left[\sec\left(\frac{\pi a}{2w}\right)\right]}^{1/2}\left[1-0.025{\left(\frac{a}{w}\right)}^{2}+0.06{\left(\frac{a}{w}\right)}^{4}\right],$$

where *a* is half of the center crack length and *w* half of the specimen width.

### 4.3. Residual Stress Effect on Corner Crack Growth

**Figure 8:**
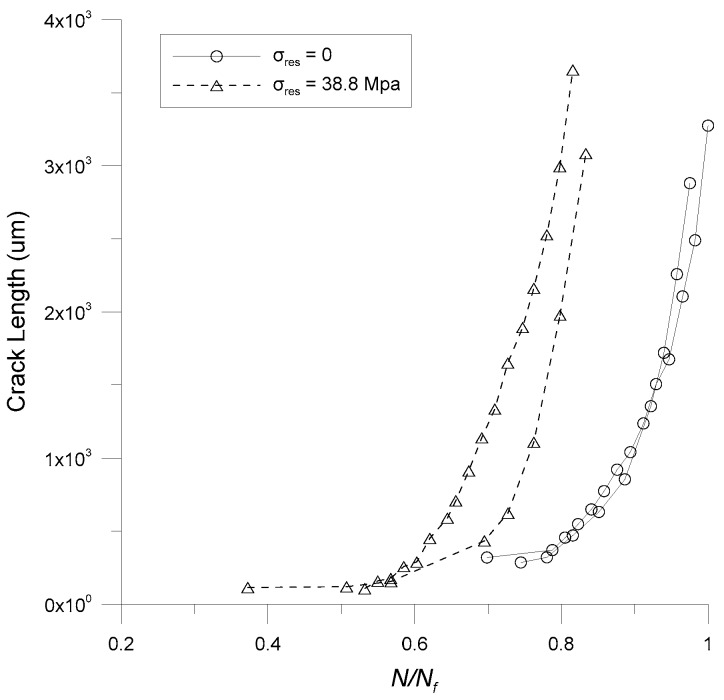
Crack length as a function of normalized fatigue life.

Here, the effect of a uniform residual stress field on the growth rate of a naturally initiated corner crack in the modified rotary specimen is investigated. A tensile residual stress of 38.8 MPa is induced in the specimen by applying an overload on the four-point bending fixture, as mentioned previously. Figure 8 shows the crack lengths of specimens with and without residual stress as a function of the number of cycles normalized by the maximum fatigue life. As shown in Figure 8, the corner crack initiates quickly on the specimen with residual stress. In addition, the fatigue life of the specimen with residual stress is about 17% shorter than that of the specimen without residual stress.

**Figure 9:**
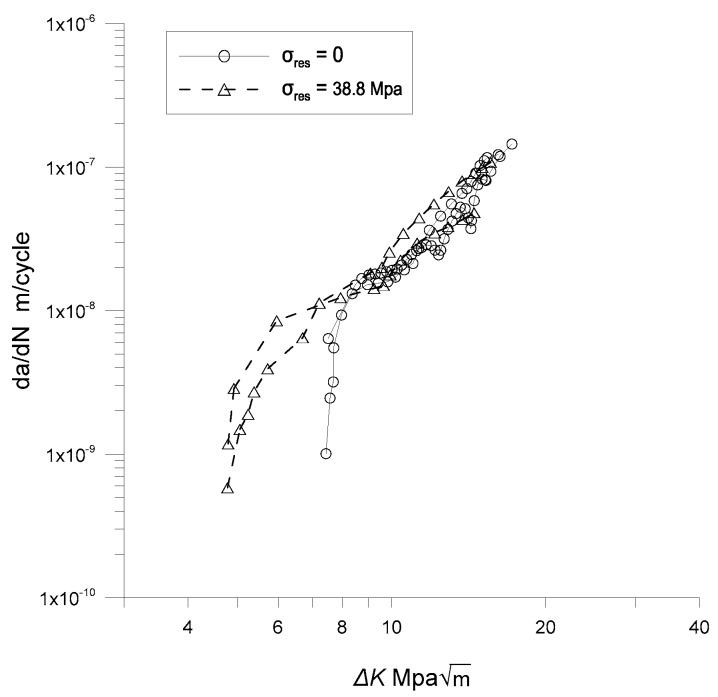
Crack growth rates of specimens with and without residual stress.

Figure 9 compares the crack growth rates of the specimens, both with and without residual stress. As shown in this figure, the corner cracks with the residual stress grow even in the low stress-intensity range, where corner cracks without residual stress do not propagate. However, the residual stress effect seems to disappear as the stress intensity increases beyond 10 MPa$\sqrt{m}$. Note that the maximum tensile residual stress occurs on the surface of the specimen and decreases with the depth of the specimen because it is introduced by bending. Therefore, the crack tip range affected by the residual stress diminishes as the crack grows. Hence, the residual stress mainly affects when the crack is small compared to the specimen. As the crack size increases, its tip portion affected by the residual stress field decreases, and this consequently reduces its effect on the crack growth rate.

**Figure 10:**
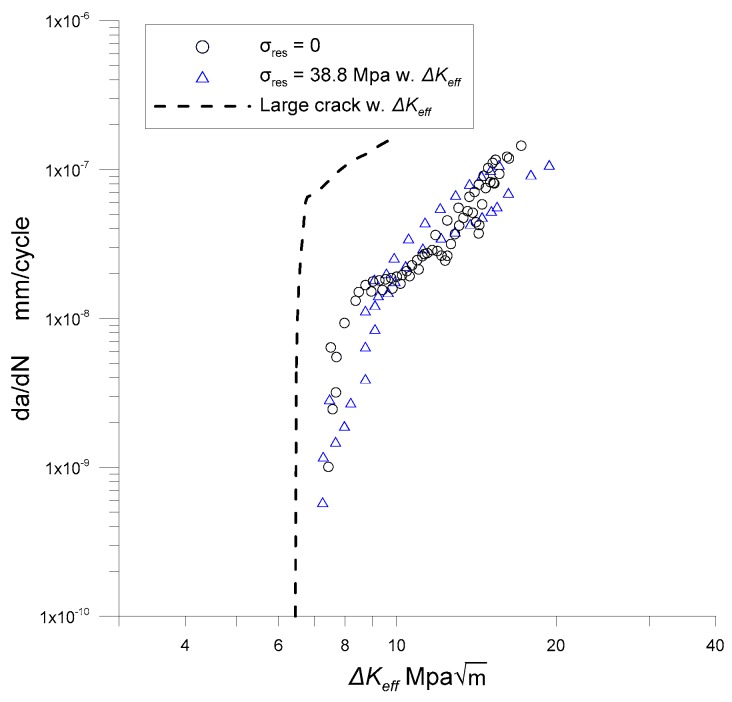
Growth rates of corner cracks along ∆*K_eff_*.

To exclude the effect of the residual stress, the principal of superposition is utilized, as indicated in Equation (20). The stress intensity induced by the residual stress, *K_res_*, is subtracted from the maximum stress intensity *K*_max_. Figure 10 shows the results of excluding the residual stress, indicated by triangular markers. As shown in Figure 10, the crack growth rates of the specimens with residual stress are in good agreement with those without residual stress once the residual stress effect is excluded by the principal of superposition. In addition, the growth behaviors of the corner cracks can be conservatively estimated with the long crack excluding the crack closure effect as shown in Figure 10 with dashed lines.

(20) $$\Delta K_{eff}=K_{\max}-K_{res}$$

## 5. Summary and Conclusions

In this study, the effect of a tensile residual stress on the growth behavior of a naturally initiated corner crack is investigated with a rotary bending specimen made of Aluminum 6061-T6. For this purpose, the middle region of a cylindrical specimen, which is the shape typically used for rotary bending tests, is modified to be square-beam shaped. A uniform residual stress is introduced on the surface of the specimen by simply applying an overload bending moment using four-point bending fixtures. The applied residual stress is evaluated based on the inelastic bending theory. 

The test results show that the growth behavior of a naturally initiated corner crack is similar to that of a long through crack. The corner crack, however, grows in the low stress intensity range below the threshold of the long crack growth rate obtained from a CCT specimen. This tendency could be attributed to the crack closure effect. It is shown that the growth of the corner crack could be conservatively estimated with the long crack data in which the crack closure effect is excluded utilizing the principle of superposition. 

The presence of tensile residual stress expedites the initiation and growth of a corner crack in a low stress intensity range. The residual stress effect, however, could be also excluded effectively using the principal of superposition. When the stress intensity caused by the residual stress is subtracted from the maximum stress intensity, the growth behavior of the corner crack with residual stress is in good agreement with that of a crack without residual stress. Therefore, the growth behavior of a naturally initiated corner crack can be estimated with long crack data regardless of the presence of residual stress. 

## Figures and Tables

**Table 1:** Coefficients of polynomial obtained from data regression.

Specimen Number	Coefficients of Polynomial for Moment Function		
	*C*_1_ (× 10^8^)	*C*_2_ (× 10^8^)	*C*_3_ (× 10^8^)
1	−4.30	6.72	−8.14
2	−3.95	6.36	4.68
3	−3.72	6.25	5.22
4	−3.88	6.32	5.33
5	−3.83	6.39	7.02
6	−3.89	6.30	5.72
7	−4.01	6.39	−1.52
8	−4.02	6.47	7.78
9	−3.99	6.45	−3.07
Average	−3.95	6.40	1.89

**Table 2:** Specimen schedule for crack growth tests.

Case num.	Specimen num.	Overload Moment (N-mm)	Residual Stress (Mpa)	Cyclic Stress (Mpa)	Cyclic Loading Frequency (Hz)
1	1	-	-	145.7	33
	2				
2	3	20,990	38.8	145.7	33
	4				
